# High-frequency oscillations arise through distinct mechanisms in models of α- synucleinopathy and channelopathy-induced epilepsy

**DOI:** 10.21203/rs.3.rs-10385398/v1

**Published:** 2026-08-13

**Authors:** Rahil N. Vaknalli, Kwaku Addo-Osafo, Vishnu Shandilya M C, Kaylin Hwang, Mariane Vicente, Anatol Bragin, Keith Vossel

**Affiliations:** Department of Neurology, David Geffen School of Medicine at UCLA, Los Angeles, CA, United States

**Keywords:** High-frequency oscillations, Epilepsy, Biomarker, Hyperactivity, Alzheimer’s disease, Dementia with Lewy Bodies

## Abstract

**Background:**

Pathological high-frequency oscillations (HFOs), widely researched in epilepsy, are increasingly being investigated as potential biomarkers of network hyperexcitability in neurodegenerative disorders. This interest is driven by growing evidence of comorbidity between epilepsy and neurodegenerative diseases, particularly Alzheimer’s disease (AD) and dementia with Lewy bodies (DLB), suggesting that these HFOs could be useful biomarkers in these disorders. Patients with AD and mouse models of AD exhibit elevated HFOs during neurophysiology recordings, but whether HFOs occur in DLB remains unknown.

**Methods:**

To address this question, we quantified cortical HFOs within the spectral range of 250–500 Hz and interictal spikes using surface electroencephalography recordings in a transgenic model of α-synucleinopathy that exhibits features of DLB (A53T mice) and a model of channelopathy-induced epilepsy (*Kcna1*−/− mice).

**Results:**

Both A53T and *Kcna1*−/− mice exhibited significantly more HFOs than their respective control mice across all vigilance states. A53T mice exhibited more HFOs and interictal spikes than *Kcna1*−/− mice, and the HFOs in A53T mice had higher frequencies and longer durations than those in *Kcna1*−/− mice. Additionally, HFOs were most prevalent during non-REM sleep in A53T mice, coinciding with the vigilance state in which interictal spikes were most frequent in this model. However, they did not consistently co-occur with interictal spikes or seizures, and HFO counts did not correlate with interictal spike counts during 10-minute recording epochs.

**Conclusions:**

Our findings indicate that HFOs likely arise from different neuronal populations and are generated through distinct mechanisms in these two models. Importantly, this work shows that HFOs are an electroencephalographic abnormality in a model of DLB and supports their potential as a new biomarker for neurodegenerative disorders.

## BACKGROUND

High frequency oscillations (HFOs) in the range of 80–200 Hz (ripples) and 250–500 Hz (fast ripples) can be recorded from both cortical and deeper structures in the normal and epileptic brain. Although the exact mechanisms underlying HFO generation is still debated, they are field potentials thought to represent transient periods of neuronal synchrony and to play important roles in normal and pathological brain function. Normal ripples may reflect synchronous inhibitory postsynaptic potentials by subsets of interneurons, whereas fast ripples are associated with pathology and considered biomarkers of epileptogenic tissue, where they coincide with ictal and interictal spikes in patients with epilepsy and animal models of mesial temporal lobe epilepsy (Levesque et al., 2015). Increasing attention has focused on HFOs in other neurological disorders, including Alzheimer’s Disease (AD). One group has demonstrated HFOs with similar characteristics in both mouse models of AD and epilepsy (Lisgaras and Scharfman, 2023). More recently, HFOs resembling those in epilepsy have also been detected non-invasively in patients with AD using magnetoencephalography, where ripple and fast ripple rates were elevated compared with controls and were differentially modulated by the anti-seizure medication levetiracetam (Shandilya et al., 2025). In individuals with Down syndrome, who almost universally develop AD pathology, fast ripples recorded using wideband scalp polysomnography emerge prior to the onset of dementia and myoclonic epilepsy, suggesting HFOs may mark epileptogenesis before clinical symptoms appear (Lisgaras et al., 2025). It remains unknown whether HFOs function as similar biomarkers of epileptogenesis in synucleinopathies, where tau and α-synuclein interactions contribute to neurodegeneration (Teravskis et al., 2018; Vacchi et al., 2020).

Increasing evidence has shown a vicious cycle between epilepsy and neurodegenerative disorders. Several studies suggest a cycle whereby endogenous tau enables network hyperexcitability which then triggers homeostatic mechanisms to phosphorylate tau, leading to tau aggregation in the brain (Chang et al., 2021; Holth et al., 2013; Liu et al., 2016; Thom et al., 2011). Aberrant excitatory network activity occurring upstream may be contributing to cognitive decline along with phosphorylated tau and amyloid-β accumulation (Hwang et al., 2022; Palop and Mucke, 2009). Seizures in patients with dementia with Lewy bodies (DLB) are estimated to be ten times higher than age matched controls with the cumulative probability of developing seizures after disease onset nearly 15% in patients with DLB (Beagle et al., 2017). Seizures in DLB are believed to accelerate cognitive decline. Even in the case of Parkinson’s disease, there has been increasing evidence in patients that link high frequency oscillations greater than 100 Hz as a pathophysiological abnormality. For instance, Johnson and colleagues characterized local field potentials recorded from deep brain stimulation electrodes in patients with Parkinson’s disease and found a prominent 200–300 Hz oscillatory peak during the parkinsonian state that was attenuated by dopaminergic medication (Johnson et al., 2021). Although traditionally α-synuclein aggregation in DLB is thought to result in pre-synaptic deficits that could then contribute to network hyperexcitability in epilepsy, the alanine-53-threonine (A53T) mutation, which is linked to familial Parkinson’s disease and other parkinsonian disorders, is sufficient to impair post-synaptic function in a tau- dependent manner prior to neurodegeneration and α-synuclein aggregation (Teravskis et al., 2018). Recent work has revealed electroencephalogram (EEG) abnormalities in mice expressing the human α-synuclein A53T mutation including EEG slowing, interictal epileptiform spikes, and myoclonus that precede and coincide with synaptic and cognitive deficits through a tau-based mechanism (Peters et al., 2020). However, HFOs can outnumber and even mask interictal spikes in AD and epilepsy models, and therefore wideband recording approaches may be needed for detecting the full extent of hyperexcitability (Lisgaras et al., 2026).

In addition to amyloid-β and tau’s involvement in network hyperexcitability, voltage gated sodium and potassium channels are critical components of neuronal signaling that can be influenced by accumulation of these proteins (Tzavellas et al., 2024). Tzavellas and colleagues have shown that overexpressing amyloid-β in mice can reduce Kv4.2 channels in CA1 pyramidal neuron dendrites and that tau was required (Tzavellas et al., 2024). Given the cross talk involved in amyloid-β, tau and voltage gated channels, we were interested in elucidating whether network hyperexcitability could occur through similar or different mechanisms in neurodegenerative disorders versus channelopathies. Motivated by the potential of HFOs as a new electroencephalographic biomarker for AD, we decided to explore whether HFOs can be detected in the A53T mouse model of DLB using cortical electrodes and explore whether HFOs might be like those seen in the *Kcna1*−/− mouse, a model of genetic epilepsy. *Kcna1*−/− mice, which lack the alpha subunit of the voltage gated potassium channel Kv1.1 exhibit various neurological disorders, including developmental temporal lobe epilepsy and sudden unexplained death in epilepsy (Glasscock et al., 2010; Smart et al., 1998). Although *Kcna1*−/− mice display HFOs during in vitro hippocampal slice electrophysiology (Simeone et al., 2013), little is known about cortical HFO patterns in this model or whether such HFOs resemble those observed in DLB models.

## METHODS

### Animals

In this study, we utilized two mouse models on a C57Bl/6J genetic background: a genetic model of channelopathy-induced epilepsy (*Kcna1*−/−) and a transgenic model of DLB (A53T). *Kcna1*−/− (Jackson Labs, #003532) mice lack the voltage-gated potassium channel alpha subunit Kv1.1. Mice lacking Kv1.1 experience symptoms of hyperexcitability, including episodic ataxia and myokymia. They develop spontaneous seizures and premature mortality beginning at P21. A53T mice (Jackson Labs, #006823) are heterozygous carriers of a mutant A53T missense form of α-synuclein transgene under the control of the mouse prion promoter. These mice exhibit learning deficits prior to the onset of α-synuclein aggregation and motor dysfunction, and the A53T mutant α-synuclein induces tau-dependent postsynaptic deficits mediated by glycogen synthase kinase 3β (GSK-3β) and a reduced AMPA-to-NMDA receptor ratio. For the A53T line experiments, 4 nontransgenic control mice and 8 transgenic mice were used in the age range of 6–10 months, whereas for the *Kcna1*−/− line experiments, 12 wild-type controls and 14 homozygous knockout mice were used in the age range of 1–2 months at the time of recording.

Animals were housed in a barrier facility with a 12-h light/dark cycle and ad libitum access to food and water. Following completion of breeding or experimental procedures, mice were euthanized either by carbon dioxide inhalation followed by cervical dislocation or, under deep isoflurane anesthesia, by transcardial perfusion with saline. All experimental protocols involving mice were in strict adherence to the National Institutes of Health’s Guide for the Care and Use of Laboratory Animals and were approved by the Institutional Animal Care and Use Committee of the University of California, Los Angeles.

### Intracranial EEG Surgeries

We used approximately equal numbers of male and female mice for EEG surgeries. 6 mg/kg of subcutaneous carprofen was used as an analgesic for *Kcna1*−/− mice and 3.25 mg/kg subcutaneous slow releasing buprenorphine was used as an analgesic for A53T mice. The difference in analgesic mode between the two lines was due to buprenorphine increasing the propensity for seizures in the *Kcna1*−/− line, leading to lower survival rates. Mice were anesthetized with 3% isoflurane and transferred to the stereotaxic mount with anesthesia maintained at 1.5% isoflurane. The scalp was shaved, the head secured using ear bars and ophthalmic lubricant was applied to the eyes. Four burr holes were made for screw electrode placement to place two on each side of the skull symmetrically across the midline, (two at 1 mm anterior/posterior and 1 mm medial/lateral, two at – 2 mm anterior/posterior and 2 mm medial/lateral relative to bregma). 0.1-inch screws with pre-soldered wires were implanted and secured to the cortical surface using dental acrylic (#8403, Pinnacle Technology, Lawrence, KS, USA). An 8-pin surface mount was soldered to the wires so that the posterior electrodes would record on two parietal channels with reference to the left anterior screw (#8431 -SM-C, Pinnacle Technology, Lawrence, KS, USA). The right anterior screw was used as ground. Mice from all groups had two EMG leads embedded in the nuchal muscles and insulated using dental acrylic for sleep analysis. All exposed wiring was also covered with dental acrylic. Mice recovered over 5 days before recordings, and *Kcna1*−/− mice received carprofen for an additional two days as post-operative care.

### EEG Recordings

Video-EEG with EMG was recorded with Sirenia Acquisition software (Pinnacle). Mice were housed in a cylindrical cage (10 inches diameter × 12 inches height) with ad libitum access to food and water and were tethered to a swivel commutator by a 10x gain preamplifier during recording which transmitted biological signal to the acquisition system (Pinnacle). The recording room also had a 12-h light/dark cycle like the barrier facility where mice were housed post-surgery. Traces from the left and right parietal cortices were recorded for 24 h on separate channels at a sampling rate of 2000 Hz with high and low-pass analog filters applied of 1 and 1000 Hz for the EEG signal and 1 and 40 Hz for the EMG signal. Video was recorded with the EEG data at a rate of 30 frames per second. Traces were exported to European Data Format for interictal spike analysis and were analyzed in the Pinnacle file format for sleep analysis. Analyses were conducted while blinded to genotype.

### Sleep Analysis

Sleep analysis was semi-automatically carried out using Sirenia Sleep Pro software (Pinnacle). EEG and EMG recordings were used to reliably distinguish wakefulness, rapid eye movement (REM) sleep, and non-REM (NREM) sleep. 10-second epochs were scored as wake, NREM and REM sleep based on a combination of EEG and EMG patterns as described previously (Peters et al., 2020). In brief, wakefulness was characterized by a low amplitude EEG with high EMG signal. NREM sleep was characterized by a higher-amplitude EEG signal, absence of EMG activity, and dominant delta-frequency activity. REM sleep was characterized by low-amplitude EEG with dominant theta-frequency activity. The sleep analysis was aided by the power density spectral plot feature in Sirenia, which allowed the user to look at the predominant frequencies in any given epoch. Analysis was accompanied by the video to verify movement.

### HFO Analysis

HFOs were analyzed in the range of 250–500 Hz because of prior studies indicating that range to be indicative of pathological HFOs. Bouts of wakeful, non-REM and REM epochs were exported from Sirenia Sleep Pro after sleep scoring to select a total of 10 min in each state of vigilance. Prior work indicates that 10 min is sufficient to measure HFOs since they are present from one 10-min period to the next (Lisgaras and Scharfman, 2022). HFOs were detected using an automated approach in RippleLab application on MATLAB and an algorithm developed by Staba that is optimized for fast ripples (Staba et al., 2002; Navarrete et al., 2016). After band-pass filtering the EEG signal between 250 and 500 Hz using a digital filter, the root mean square amplitude was quantified. Root mean square amplitudes greater than 5 standard deviations above the mean amplitude of the root mean square signal with a duration greater than 6 ms were selected as potential HFOs for manual review. Each animal had its own baseline for detecting HFOs as HFO analysis for each animal involved new baseline root mean square values to be calculated depending on the vigilance state. True HFOs were manually selected from the potential HFOs through the algorithm based on the criteria mentioned in previous work (Lisgaras and Scharfman, 2022; 2023). We used the time-frequency domain feature in RippleLab to look at HFO power over time and artifacts were identified by the absence of a tuned frequency in the time-frequency domain. True HFOs were well resolved with islands with a central frequency appearing on the time-frequency domain. Artifacts were excluded from the analysis.

### Interictal Spike Analysis

Interictal spikes were classified as high-amplitude deflections, less than 75 ms arising from the background and greater than 5 standard deviations from the baseline mean signal using the same technique described previously (Peters et al., 2020). Spikes were detected using Spike2 software (Cambridge Electronic Design, Cambridge, UK). In brief, 200-ms intervals of candidate spikes were extracted from bilateral EEG traces to a stereotrode channel and plotted into principal component space. K means clustering (K = 15) segregated the waveforms into different groups based on similar components. These groups formed templates that candidate spikes in the recording were compared against. Templates determined to be artifacts because of deflections longer than 75 ms were excluded from clustering. The principal components step was reiterated several times leading to a finer tuning of the templates to spike features. Lastly, artifacts and templates that were in doubt were manually reviewed.

### Statistical Analysis

Approximately equal number of male and female mice were used for the experiments, with both sexes combined in statistical analyses after showing no sex-dependent effects. All statistical analyses were carried out using Graphpad Prism version 9 (GraphPad Software, San Diego, CA, USA). The data is presented as means ± SEM, and sample sizes are provided in the figure legends. To determine if data fit a Gaussian distribution, the Shapiro-Wilk test was used. Welch’s t test was used to compare HFO data for the A53T mice, which followed a normal distribution. For *Kcna1*−/− HFO data, the Mann–Whitney test was used due to non-normal distribution. For correlation analyses Spearman’s rank correlation was used for nonparametric data and Pearson’s correlation coefficient (r) was used for parametric data. For comparisons involving more than two groups, a Kruskal-Wallis test was used for nonparametric data, followed by Dunn’s post hoc tests when significant. For parametric data involving multiple comparisons, a two-way analysis of variance (ANOVA) was used with Šidák’s multiple comparisons test.

## RESULTS

### HFOs in the parietal cortex have more distinct characteristics in A53T mice than in Kcna1−/− mice.

A53T mice exhibited HFOs in EEG recordings from the surface of the parietal cortex. In these mice, HFOs, particularly those coinciding with interictal spikes (black arrow), were more distinct than in nontransgenic control mice and stood out more clearly from the filtered background EEG data (Fig. 1A,B). *Kcna1*−/− mice also exhibited HFOs in the parietal cortex, but the morphology of the HFOs in the filtered traces was indistinguishable from HFOs in wild-type controls (Fig. 2A,B). After pooling HFOs across vigilance states, A53T mice had a higher number of HFOs than nontransgenic controls (unpaired t test with Welch’s correction, t = 3.087, df = 9.937, p = 0.0116) (Fig. 1C), whereas *Kcna1*−/− mice showed a modest increase in HFOs that was not statistically significant (Mann-Whitney test, U = 56, p = 0.0998) (Fig. 2C). HFOs in control mice likely reflect physiological activity within the 250–500 Hz range.

### HFOs in A53T mice are most elevated during NREM sleep

We next evaluated whether there are variations in HFO rates across different vigilance states, since interictal spikes are most common in NREM sleep > wakefulness > REM sleep in A53T mice (Peters et al., 2020) and pathological HFOs are a biomarker of hyperexcitability. We examined HFOs in A53T mice and *Kcna1*−/− mice, in comparison to their respective control mice, categorized based on vigilance states (Fig. 3A,B). HFO rates in A53T mice were increased during NREM sleep compared to during REM sleep (p = 0.0168) and slightly higher during NREM than during wakefulness (Fig. 3A). Compared to nontransgenic controls, HFOs occurred at higher rates in A53T mice during wakefulness and NREM sleep, but not during REM sleep (Fig. 3A). *Kcna1*−/− mice showed a genotype effect on HFO rates across all vigilance states (p = 0.0262), but there was no significant variation in HFO rates based on vigilance state (Fig. 3B). To directly compare the two models, we used a two-way ANOVA with model and vigilance state as factors (Fig. 3C). This analysis showed a significant effect for model (F (1,88) = 33.07, p < 0.0001) and vigilance state (F (3,88) = 16.13, p < 0.0001) and significant interaction (F (3,88) = 4.988, p = 0.0030), with post hoc tests showing that A53T mice had higher HFO rates during NREM sleep compared to *Kcna1*−/− mice (p = 0.0082). There was also a significant difference in the number of HFOs after pooling vigilance states together, with A53T mice exhibiting more HFOs than *Kcna1*−/− mice (p < 0.0001).

### A53T mice have more interictal spikes than Kcna1−/− mice and different HFO characteristics

Over the span of a 24-h recording, A53T mice had a much higher frequency of epileptiform discharges than *Kcna1*−/− mice as seen through the rate of discharges over a representative 10-min period (Fig. 4A) (Mann-Whitney test, U = 22, p = 0.0130). HFOs did not significantly correlate with epileptiform discharges during 10-min windows in A53T mice or *Kcna1*−/− mice (Fig. 4B–D). However, we observed clear differences in the characteristics of HFOs in A53T and *Kcna1*−/− mice. HFOs in A53T mice had a higher oscillatory frequency (unpaired t test with Welch’s correction, t = 4.067, df = 20.57, p = 0.0006) and longer duration than in *Kcna1*−/− mice (unpaired t test with Welch’s correction, t = 2.461, df = 21.30, p = 0.0225) (Fig. 4E,F).

## DISCUSSION

In this study, we demonstrate that cortical HFOs in the 250–500 Hz range occur in a model of α-synucleinopathy (A53T mice) and a model of genetic epilepsy (*Kcna1*−/− mice). These HFOs exhibit a higher rate, higher oscillatory frequency, and longer duration in the A53T mice compared to *Kcna1*−/− mice. Additionally, HFOs in the A53T mice were most frequent during NREM sleep, coinciding with the vigilance state in which epileptiform activity is also more common in this model (Peters et al., 2020). Based on this finding, NREM sleep could have a stronger role in facilitating HFO events in the A53T model than in the *Kcna1*−/− model. It is also possible that the HFOs occurring during NREM in the A53T model are more likely to be pathological than those occurring during wakefulness and REM sleep.

Distinguishing pathological from nonpathological HFOs is an active area of investigation. Some studies suggest that HFOs can be distinguished based on frequency bands with normal HFOs occurring in the ripple frequency range and pathological HFOs in the fast ripple range (Ye et al., 2024; Matsumoto et al., 2013). However, HFOs cannot be defined based solely on frequency as HFOs within the fast ripple frequency range can occur in the normal neocortex while oscillations in the ripple frequency range can occur in the epileptic dentate gyrus (Engel Jr et al., 2009). Normal HFOs are thought to reflect summated synchronous inhibitory postsynaptic potentials from interneurons. Pathological HFOs on the other hand can occur as interictal events or coincide with ictal discharges and may be associated with regions capable of generating spontaneous seizures. As the nontransgenic mice do not exhibit seizures, it is likely that most of the HFOs in the nontransgenic controls were not pathological even though they were in the fast ripple range, and that HFOs recorded from the cortical surface are a combination of normal and pathological HFOs in the various models. Additionally, the stronger association between HFOs and interictal spikes during NREM sleep in A53T mice compared to *Kcna1*−/− mice may suggest that HFOs in A53T mice are more pathological, as they more often co-occur with interictal spike emergence.

The finding that more HFOs occurred during NREM sleep in the A53T model is consistent with human and rodent data from epilepsy as described by Lisgaras and Scharfman, where HFOs could be facilitated by periods of high synchronization such as transitions from depolarizing “up” states to hyperpolarizing “down” states (Lisgaras and Scharfman, 2022). The significant difference in HFO occurrences between *Kcna1*−/− and A53T mice during NREM sleep suggests that there could be specific effects due to the A53T α-synuclein driving these differences. Peters and colleagues observed that in A53T mice, endogenous tau is a critical mediator of excitotoxicity and interictal epileptiform discharges prior to α-synuclein deposition. This is supported by the early emergence of hyperexcitability at 3–4 months of age and by the finding that tau knockdown attenuates epileptiform discharges. Potential mechanisms include tau missorting into dendritic spines driven by A53T α-synuclein, calcineurin-mediated AMPA receptor internalization, and inhibitory hippocampal circuit remodeling (Teravskis et al., 2018). Interactions between tau and GSK-3β are also important in epilepsy, where GSK-3β phosphorylates tau and its inhibition reduces tau hyperphosphorylation and exerts anti-epileptic effects against pentylenetetrazol-induced seizures in zebrafish (Aourz et al., 2019). A53T mice have dysregulated GSK-3β which could play a role in hyperexcitability through tau phosphorylation.

The *Kcna1*−/− model exhibits features relevant to temporal lobe epilepsy, where HFOs in the fast ripple frequency range have been most extensively characterized in the hippocampus and entorhinal cortex, although cortical HFOs in this model have not been well studied. In rodents, neurons generating fast ripples are not homogeneously distributed but occur in discrete clusters. Additionally, differing mechanisms contributing to epileptic activity may be present in *Kcna1*−/− mice compared to the A53T model, especially given differences in HFO frequency and duration. One study noted that fast ripples in *Kcna1*−/− hippocampal slices arise from enhanced synaptic release in CA3, leading to reduced precision in neuronal firing. However, these fast ripples remain localized to the hippocampus and are not observed in the entorhinal cortex of *Kcna1*−/− mice (Simeone et al., 2013). On the other hand, the HFOs arising in the A53T model may reflect broader synaptic and network alterations involving α-synuclein, tau, or neuroinflammation.

Overall, we propose that the elevated HFOs and elevated interictal spikes in the A53T group might result from the synaptic deficits induced through the A53T mutation, leading to pathological interactions between tau and α-synuclein. These findings suggest distinct mechanisms underlying HFO generation in epilepsy driven by channelopathy versus α-synuclein pathology, and provide insight into aberrant network activity relevant to DLB.

This study has several limitations. First, the *Kcna1*−/− and A53T cohorts were not age-matched due to differences in disease progression, and premature mortality in *Kcna1*−/− mice limited sample size and statistical power for detecting more subtle relationships. Second, cortical surface recordings may not fully capture HFOs originating in deeper structures such as the hippocampus and entorhinal cortex. This is particularly relevant for the *Kcna1*−/− model, where fast ripples have been most extensively characterized in hippocampal networks. Finally, distinguishing physiological from pathological HFOs remains challenging, as frequency-based classification is insufficient and cortical signals likely reflect a mixture of both. Future studies using depth recordings and improved approaches to classify HFOs will help further define their cellular and network origins across neurodegenerative and epileptic conditions

## CONCLUSIONS

Overall, demonstrating that HFOs are an electrophysiological abnormality in both A53T and *Kcna1*−/− mice has important implications for understanding distinct mechanisms of epileptogenesis. As noted by Scharfman and colleagues in a study of an AD model, HFOs do not necessarily arise from epilepsy or from AD pathology, as they were observed in mice without overt seizures or AD pathology (Lisgaras and Scharfman, 2023). This concept could be in line with the idea of the latent phase theory in epileptogenesis where HFOs and related molecular and cellular events emerge during a seizure-free latent period and may contribute to the development of spontaneous recurrent seizures in the chronic phase (Mokhothu and Tanaka, 2021).

Importantly, our findings support the idea that HFOs may represent a shared biomarker of network hyperexcitability across both neurodegenerative and epileptic disorders, including DLB-related pathology. Defining the causal mechanisms underlying HFO generation in synucleinopathies compared to channelopathies may ultimately inform early biomarkers and therapeutic strategies for these disorders.

## Figures and Tables

**Figure 1:**
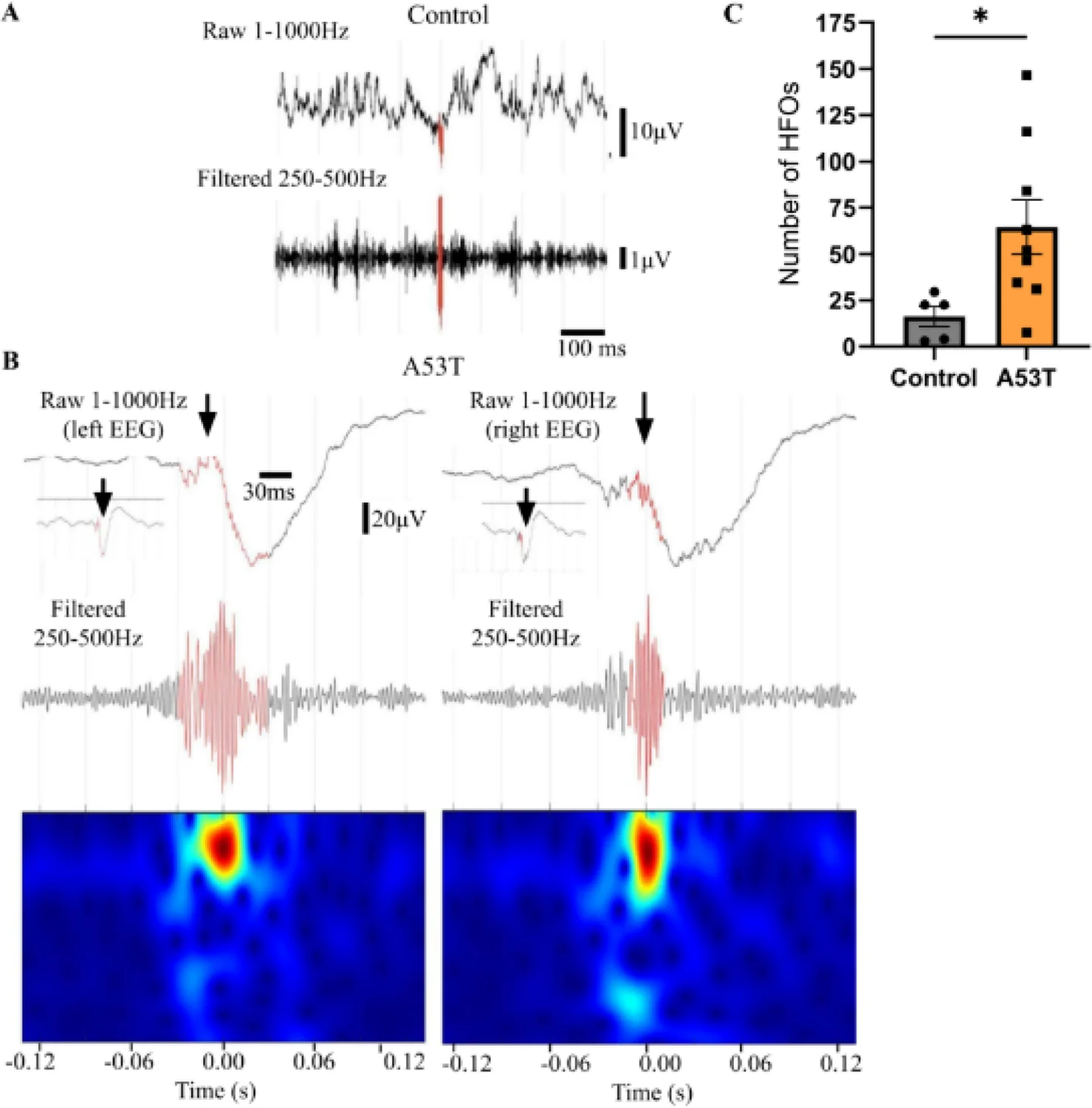
HFOs in parietal cortex of A53T mice are distinct from those in control mice. **A, B**. Shown are representative EEG recordings from the surface of the parietal cortex of a nontransgenic control mouse **(A)**and an A53T mouse **(B)**. The HFO in the A53T mouse coincides with an interictal spike (arrow). The top traces are raw (unfiltered traces) and the bottom traces are band-pass filtered at 250–500 Hz. HFOs can be seen in both EEG channels for the A53T mouse Note that both control and A53T mice exhibit HFOs (red traces) but the HFO in the A53T mouse is more robust. Frequency-time plots for both channels show minimal artifacts with a “blob-like” appearance. **C**. HFOs across all vigilance states (wake, NREM and REM sleep) were more frequent in A53T mice (n=9) than in controls (n=5) (unpaired t test with Welch’s correction, t = 3.087, df = 9.937, *p = 0.0116.). Note that each animal had 30 min of EEG data analyzed, with 10 min of EEG data in each state.

**Figure 2:**
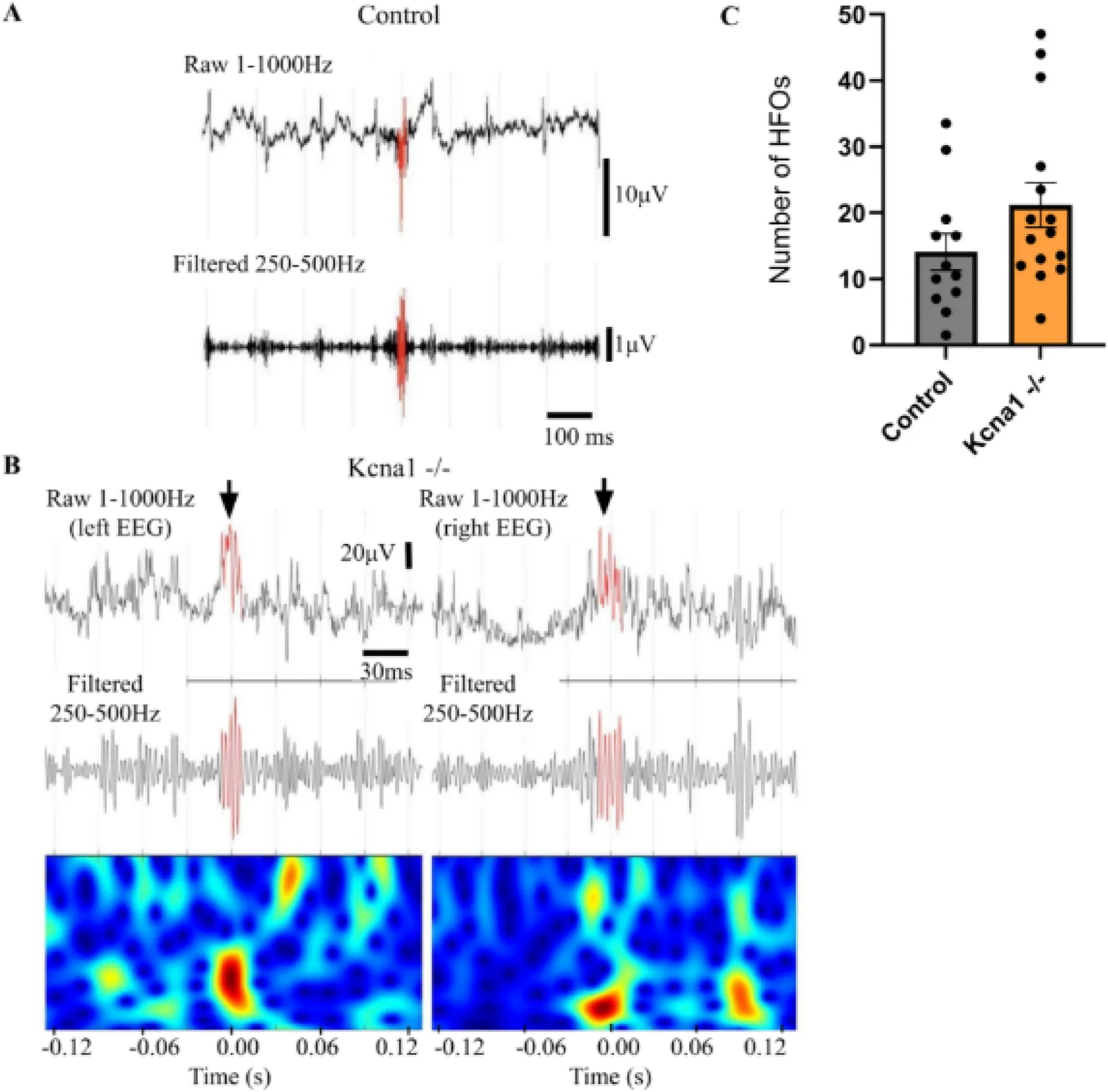
HFOs in *Kcna1*−/− mice. **A**. A representative EEG recording from the surface of the parietal cortex of control mice during NREM sleep. **B**. Same as in A but in a *Kcna1*−/− mouse. HFOs can be seen in the red trace in both the control and *Kcna1*−/− mouse. Frequency-time plots for both channels show slightly more noise compared to A53T mice. **C**. Total number of HFOs across all vigilance states in control (n=12) and *Kcna1*−/− mice (n=15) from three 10-min periods over the course of a 24-h recording, with each 10-min period from a different vigilant state. The total number of HFOs trended towards being more frequent in *Kcna1*−/− mice (Mann-Whitney test, U = 56, p = 0.0998).

**Figure 3:**
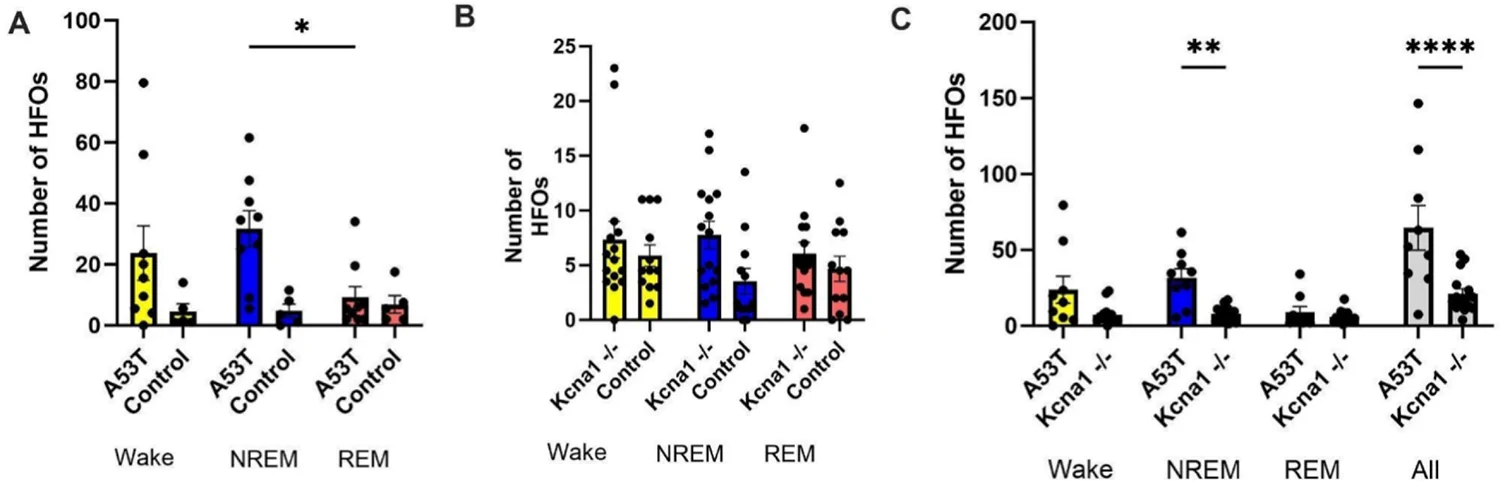
Comparison of HFOs from different vigilance states in A53T mice and *Kcna1*−/− mice. **A**. Number of HFOs during Wake, NREM and REM sleep in A53T mice. A two-way analysis of variance (ANOVA) with genotype and vigilance state as factors showed a significant effect for genotype (F (1,36) = 9.409, p = 0.0041) but not vigilance state (F (2,36) = 1.292, p = 0.2871) and no interaction (F (2,36) = 1.934, p = 0.1592). Post hoc tests revealed a significantly greater number of HFOs during NREM sleep vs REM sleep in A53T mice (*p = 0.0168). **B**. Same as in A but in *Kcna1*−/− mice. A two-way ANOVA showed a significant effect for genotype (F (1,75) = 5.146, p = 0.0262) but not vigilance state (F (2,75) = 0.5162, p = 0.5989) and no interaction (F (2,75) = 0.8019, p = 0.4523). Although post-hoc tests showed a trend toward a higher number of HFOs in NREM sleep in the *Kcna1*−/− mice than controls (p = 0.0639). **C**. Number of HFOs during Wake, NREM and REM across models (A53T and *Kcna1*−/−) with all vigilance states combined. A two-way ANOVA showed a significant effect for model (F (1,88) = 33.07, p < 0.0001), vigilance state (F (3,88) = 16.13, p < 0.0001) and significant interaction (F (3,88) = 4.988, P = 0.0030). Post hoc multiple comparisons test showed significantly elevated HFOs during NREM sleep in A53T mice than *Kcna1*−/− mice (**p = 0.0082) and a greater number of HFOs in A53T mice than *Kcna1*−/− mice with all vigilance states combined (****p < 0.0001). HFO numbers during Wake (p =0.1239) and NREM (p = 0.9905) were not different across models.

**Figure 4:**
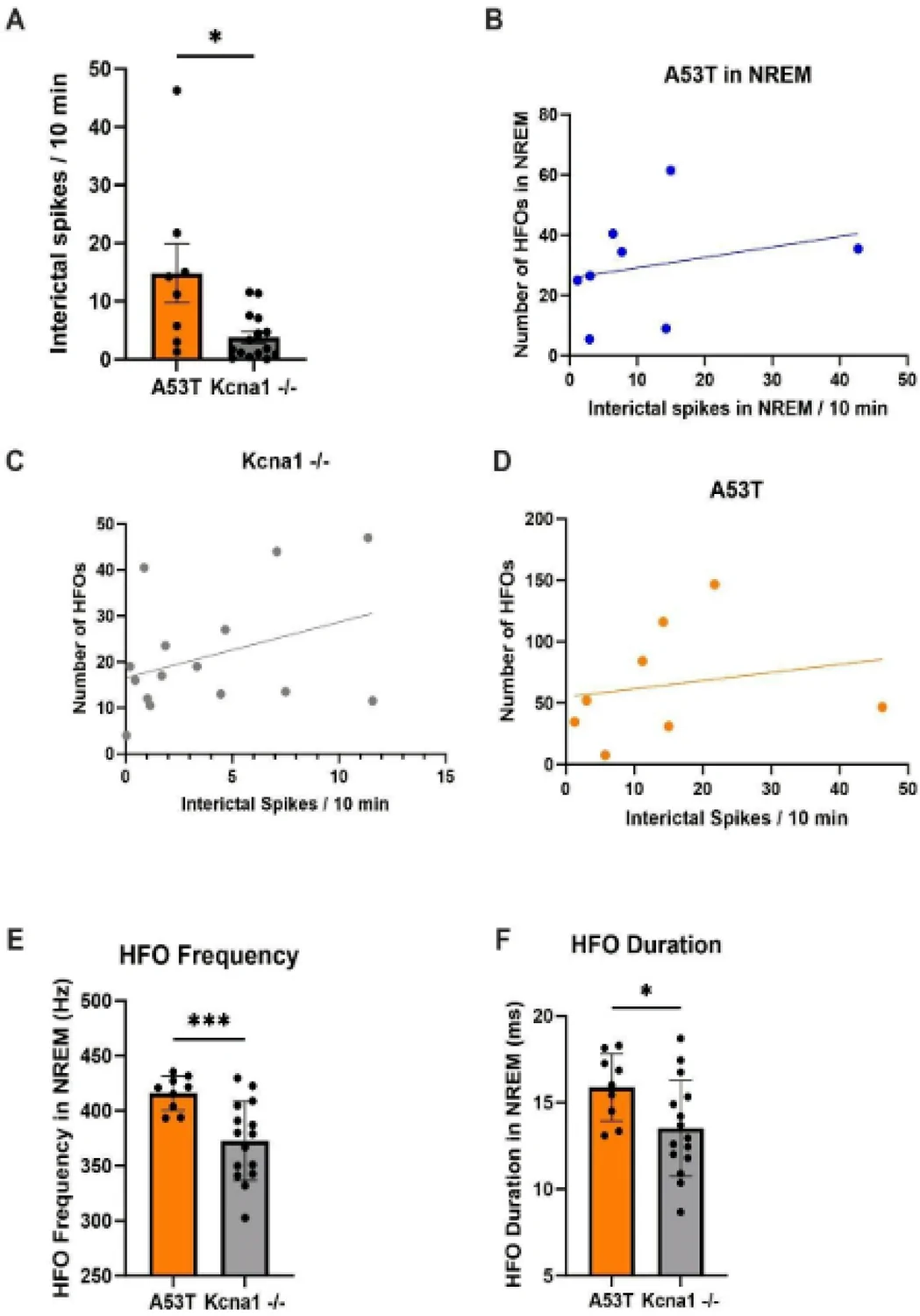
Interictal Spikes and HFO characteristics in A53T and *Kcna1*−/− mice. **A**. Number of interictal spikes in 10-min periods in A53T and *Kcna1*−/− mice across all vigilance states. A53T mice (n=8) had a significantly higher number of interictal spikes than *Kcna1*−/− mice (n = 15) (Mann-Whitney test, U = 22, *p = 0.0130) **B**. Correlation of interictal spikes and HFOs in A53T mice during NREM sleep, since A53T mice showed elevated HFOs during this state (Spearman r = 0.5714, p = 0.1511). **C**. Same as in B but in *Kcna1*−/− mice pooled during all vigilance states (Spearman r = 0.2770, p = 0.3151). **D**. Correlation of interictal spikes and HFOs in A53T mice across all vigilance states (Spearman r = 0.2029, p = 0.6299). **E**. HFOs in *Kcna1*−/− mice had a significantly lower frequency than HFOs in A53T mice (unpaired t test with Welch’s correction, t = 4.067, df = 20.57, ***p = 0.0006). **F**. Same as in E but for HFO duration in the two models. *Kcna1*−/− mice have significantly shorter HFOs than A53T mice, (unpaired t test with Welch’s correction, t = 2.461, df = 21.30, *p = 0.0225).

## Data Availability

The datasets generated and/or analyzed during the current study are available from the corresponding author upon reasonable request.
